# Comparing the effects of infant maternal and sibling separation on adolescent behavior in rats (*Rattus norvegicus*)

**DOI:** 10.1371/journal.pone.0308958

**Published:** 2024-08-16

**Authors:** Shane R. McClafferty, Claudia Paniagua-Ugarte, Zoe M. Hannabass, Pamela A. Jackson, Dayna M. Hayes

**Affiliations:** 1 Radford University, Radford, VA, United States of America; 2 Virginia Polytechnic Institute and State University, Blacksburg, VA, United States of America; Children’s Hospital Affiliated of Zhengzhou University: Zhengzhou Children’s Hospital, CHINA

## Abstract

Maternal separation in early life has been observed to have lasting, detrimental effects that impair personal and social development and can persist into adulthood. Maternal separation during infancy can be most detrimental during adolescence, leading to long-term adverse effects on development and social behavior. This research study compared the effects of sibling and maternal separation in infancy on anxiety, sociability, or memory later in adolescence (postnatal day, PND, 50–58) in male and female Long-Evans Rats (*Rattus norvegicus*). Rat pups were semi-randomly assigned into eight conditions for daily isolation (PND 1–14). The groups were separated by the duration of isolation between 15 minutes (control group) or 180 minutes (experimental group) and the sex of the rat. They were also separated by comfort conditions with the dam present in an adjoining cage versus not present and siblings present or not present during isolation. The result was a 2 (15-min vs. 180-min) x 2 (dam vs. no dam) x 2 (single vs. grouped) x 2 (male vs. female) design. Once pups had reached adolescence (PND 50), researchers tested for differences in anxiety, activity, and social behavior using elevated plus-maze, open field habituation, a three-chamber social interaction, and a social discrimination task. Results indicate that longer isolation was more stressful and caused lower body weight. The female rats showed more anxious behavior in the open field but only if they were in the shorter isolation group. Social interaction showed that the rats isolated with the dam had different effects of isolation. In males, shorter isolation with the dam increased sociability but decreased sociability in females. These complicated findings may be due to the effects of inoculation, which describes how moderate stress combined with comfort may produce adaptation or immunity to stress and affect males and females differently.

## Introduction

During early development, stress caused by disruptions in maternal care and comfort can have lasting effects on both rats (*Rattus norvegicus*) [1–4] and humans (*Homo sapiens*) [5, 6]. Studies show that maternal separation during infancy and early development can lead to increased anxiety, less exploratory behavior, and decreased social play in adolescent mice (*Mus musculus*) and rats [1–4, 7–12]. Other studies have found impairments in memory, slower learning, and alterations in cognitive development in humans and animals [1, 13–17]. These results imply that early stress contributes to prolonged social and psychological issues like anxiety disorders, attachment disorders, and cognitive deficits. However, in an analysis of the time course of behavioral changes following early life stress, deficits in affect and activity were only found at about postnatal day (PND) 40–60 [7], which is roughly the adolescent period [3]. The adolescent period is more vulnerable to negative impacts and is a time when emotional reactivity and sociability increase [18].

One possible explanation is that early stress in infancy may lead to exaggerated responses to stress in adulthood [10, 19]. Severe stress during infancy has been linked to the development of negative feedback loops in the hypothalamus-pituitary-adrenal axis (the cortisol response) [1, 20], and cortisol antagonists reduce the stress response (ultrasonic vocalizations) during isolation [21]. However, these effects may be moderated by comfort factors during infancy, like being licked by the dam [20, 22]. Maternal licking has also been found to have different effects on male and female rats [20, 22, 23]. Maternal licking in male rats is associated with increased vasopressin receptors [22], which is associated with anxiety and sociability [23]. However, maternal licking in female rats is associated with increased oxytocin instead of vasopressin [22], which is associated with decreased anxiety but increased aggression [20]. Similar comfort factors like smelling the dam and touching or smelling the siblings can be manipulated experimentally during early isolation stress [10, 21].

Models for early stress include maternal separation (MS), sibling separation (SS), and early handling (EH) [10, 24]. An MS procedure is the separation of pups from the dam. SS involves the separation of pups from the other members of their litter, and the SS procedure usually also involves MS. EH is the researcher touching or moving a rat early in development and can have similar effects to MS [3]. However, few studies compared the difference between SS and MS, especially not within the same litter. The present study will use a novel MS and SS apparatus on PND 1–14 to test the behavioral effects in adolescence (PND 50–58). The apparatus allowed multiple siblings to be isolated or grouped together while remaining near the dam (although not in contact) or without the dam.

Previous studies have shown that rats present with detrimental behavior caused by MS and SS on tasks such as elevated-plus-maze (EPM), open-field exploration (OF), social interaction (SI), and social memory or discrimination (SD) [3, 4, 14]. For example, Lukas et al. [14] used a similar MS procedure to the present study (3-hours of separation from PND 1 to 14) and a control group without EH. They found that the MS rats tested in adolescence did not differ from the control rats using 30 or 60-min intervals between presentations of the conspecific rats. However, when tested as adults, the MS rats failed to discriminate between the same and the novel conspecific rats. In general, longer isolation led to detrimental effects on anxiety, SI, and SD. Similarly, Jin et al. [3] applied an MS procedure to rats for their first twenty-one days after birth for 3-hours or 15-min per day. They found that longer maternal separation decreased anxiety-like behaviors and locomotor activity during adolescence.

Alternatively, Kambali et al. [4] used both MS and SS for six hours daily from day 4–14 while controlling for EH (6-hours vs. zero handling). When the male rats were tested in adolescence, they exhibited increased anxiety-like behaviors, increased locomotor activity, and decreased body weight. On the SD task, Kambali et al. [4] found that the MS rats spent more time with the familiar rat, whereas the control rats spent more time with the new conspecific. They suggested that this difference may be evidence of social inflexibility in the MS-stressed adolescent rats. However, they also found enhanced spatial learning on the eight-arm radial maze for rats separated for six hours compared to no separation, but retention memory (30 days later) of the task was not different between the two groups.

Given these studies, 180-min is a long enough isolation time to produce behavioral deficits [3, 14]. A 15-min control group is within a normal time frame for the dam to leave its pups to find food, and anything longer than an hour is considered severe isolation [25]. Rats with no early handling (minimal human interaction) have been shown to exhibit much lower anxiety than both 15 or 180-min MS groups [3]. Human touching alone can drive these differences instead of time away from the dam. In fact, human tickling paradigms, even in older rats, have yielded both positive and negative effects on stress levels [26]. Thus, the 15-min group was considered the best comparison group because it allowed human interaction to be the same across our separation conditions.

Based on results from these studies, it is expected that the rats isolated without the dam and for a more extended period of time (180-min vs. 15-min separation) will exhibit detrimental behavioral effects compared to the shorter time. Given that other studies with SS have found significant differences, SS is expected to lead to similar or worse behavioral effects than MS in the current study [4, 10]. Rats isolated without the dam or siblings, especially without both, for 180-min will exhibit more anxiety-like behavior and changes in activity than those isolated for only 15-min. In the SI and SD tasks, the experimental rats isolated without the dam or siblings are expected to have less sociability and less preference for the new rat in SD. It is also anticipated that experimental groups will have stunted growth in both sexes (although males should weigh more than females throughout).

## Material and methods

### Subjects

**Fig 1:**
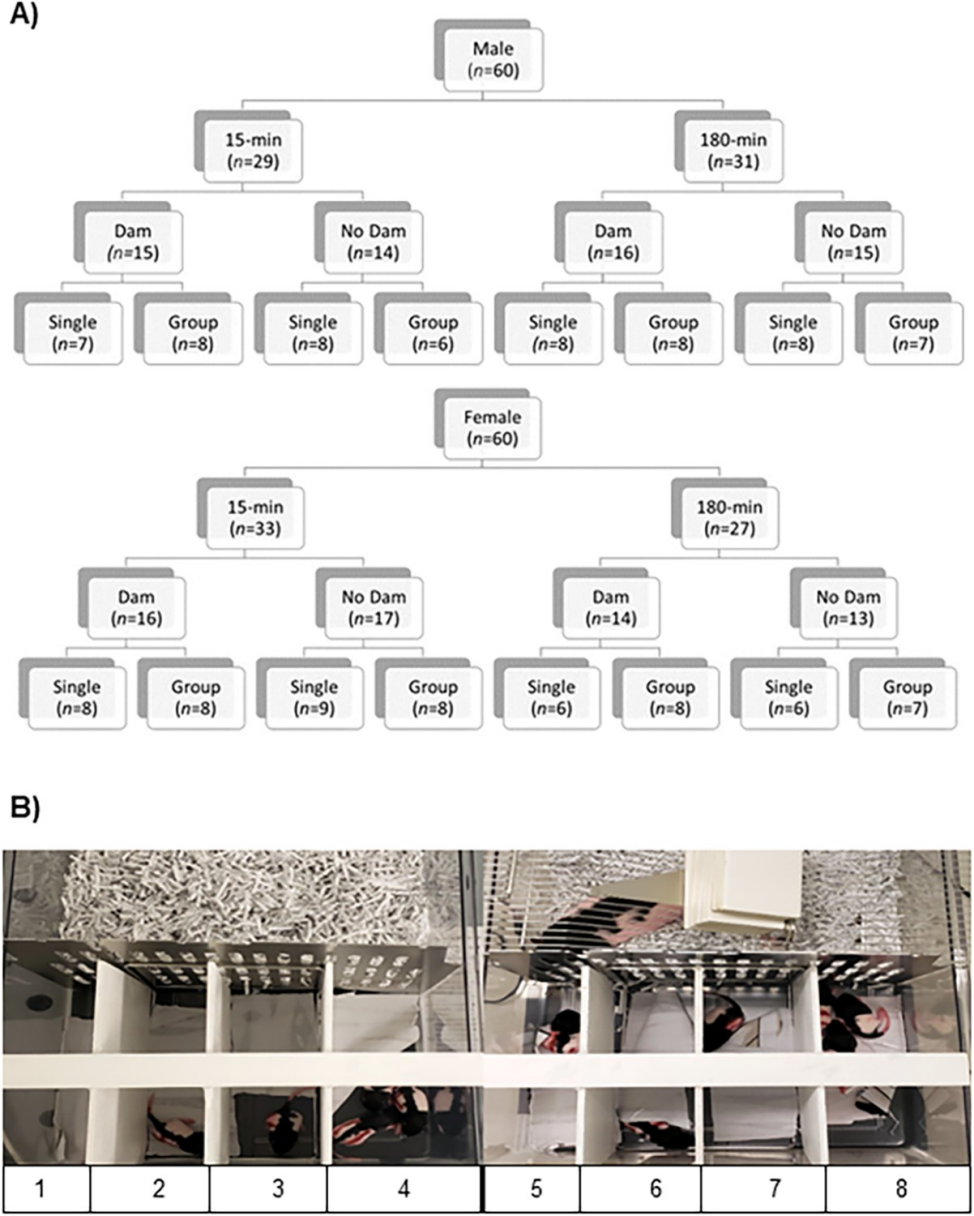
Experimental design. A) The design was a 2 (male vs. female) x 2 (180-min vs. 15-min isolation period) x 2 (dam present vs. no dam present) x 2 (single isolation with no siblings vs. grouped isolation with siblings). The number of animals per group (*n*) corresponds to the number of rats on postnatal day (PND) 50 for the elevated-plus maze task. B) The left image in the photograph depicts rat pups assigned to be housed without the dam on the opposing side of the barrier (1–4). The right image depicts rat pups isolated with the dam present on the other side of the barrier during the separation (5–8). In both images, the bottom half of the barrier is divided into four equal sections. The first three sections, from left to right, housed rat pups without siblings (1–3 and 5–7). The fourth section housed pups with at least one sibling during the isolation phase (4 and 8). Both images in B are reprinted under a CC BY license with permission from Pamela A. Jackson, 2021.

**Fig 2:**
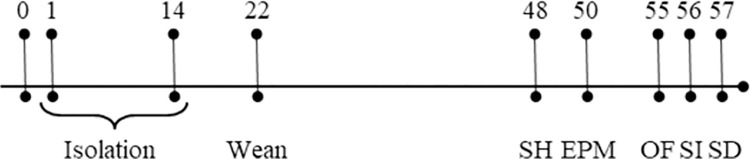
Timeline of experiment. The rat pups were born on postnatal day zero (PND 0) and underwent their assigned separation procedure from PND 1–14. The rat pups were weaned from the dam on PND 22 and housed in same-sex groups until PND 48 when they were single-housed (SH). All rats completed the elevated-plus-maze task (EPM) on PND 50. Some rats completed the open-field task on PND 55, and some on PND 56. The social interaction (SI) task was completed exactly 24 hours after OF, and the social discrimination (SD) task was completed exactly 24 hours after SI.

Breeding procedures for this study consisted of pairing two sister Long-Evans rats (*R*. *norvegicus*) with one genetically unrelated male Long-Evans rat. Six harem pairings produced pups from each sister dam (a two-litter cohort). An additional five pairings resulted in pups from only one of the sister dams. One litter of a cohort was assigned to the 180-min isolation group and the other to the 15-min group (see Fig 1A). Every litter was culled down to twelve pups (six female and six male) on the day after birth, postnatal day one (PND1), and were then otherwise randomly assigned to conditions. A timeline is provided in Fig 2. The first litter (n = 8) was dropped as it was the pilot litter and experienced somewhat different procedures. An additional seven rats were dropped at various stages of the study due to environmental and health issues.

For identification purposes, animals were marked daily using a red permanent marker along the lateral portion of their body until PND 22. After PND 22, the animals were marked on the tail every three days for identification rather than the body. Between PND 22 and PND 30, the data sheets indicating the separation manipulations for each litter of rat pups were stored separately in order to ensure that researchers collecting the behavioral data were blind to the conditions each rat experienced. Weights of the animals were collected every three days from PND 0–49. From PND 50 until euthanasia, weights were only collected on the days that behavioral tasks were conducted.

This research project was approved by the Institutional Animal Care and Use Committee (IACUC) at Radford University, and all procedures were conducted according to approved methods. Animals were housed in a temperature-controlled (22˚C) and humidity-controlled (~60%) vivarium on a 12/12 light/dark cycle (lights on at 0800) with access to food and water except where noted. Body weights were taken consistently throughout the experiment as a measure of overall health and growth. All animals were treated in accordance with the applicable portions of the Animal Welfare Act and the National Institute of Health’s “Guide for the Care and Use of Laboratory Animals” (8^th^ Edition, National Research Council of the National Academies).

### Isolation

For this experiment, rat pups were weighed and semi-randomly assigned to experimental groups on PND 1. The litter was assigned to 180 minutes (experimental group) or 15 minutes (control group) of isolation beginning on PND 1 through 14 (see Fig 2). Each litter of pups was then separated such that half the rats were isolated via a buddy barrier separating them from the dam in the other half of the cage, and half did not have the dam present as she was placed in a separate room. Each of the dam-present and the dam-not-present groups was divided such that half of the pups were housed individually, and half of the pups were isolated with one or two siblings. The apparatus can be observed in Fig 1B. In addition, each group consisted of female and male rats to assess sex differences. The result was a 2 (180-min vs. 15-min) x 2 (dam vs. no dam) x 2 (single vs. siblings) x 2 (male vs. female) design (see Fig 1A). All rats had the same human handling time regardless of condition.

At the beginning of each isolation session, the dam was separated from the pups. Then, the pups were placed in two cages lined with a heating pad to prepare and transport them. Identification markings were renewed every day, but the pups were only weighed every third day. Each cage of pups was then transported to the room with the buddy-barrier cage that would contain the dam or the room with the empty buddy-barrier cage, and the rats were placed in the cages based on their markings. Isolation sessions occurred in dim white light and white noise. All buddy barrier cages were placed on heating pads to maintain the temperature of the pups between 29-34° C (302.15–307.15°K) during isolation. The temperature was accurate at the beginning of each isolation period and adjusted every 30–45 minutes to maintain consistency. Behavioral data collection during isolation occurred approximately 10–15 minutes after being placed in isolation. The rats in the longer isolation were also observed at approximately 40-min intervals, adding up to four or five observations. In addition to recording and adjusting the temperature, behavioral measurements were also recorded, including the location of the pups in relation to the adjoining cage, whether they were in contact with their siblings, and their activity. Activity observations included whether the pups were active, pacing/walking, or grooming.

At the end of each isolation session, all pups were placed into separate holding cages, and they were transported back to the colony room and placed into the home-cage with their dam. Rat pups were weaned from the dam on PND 22 and maintained in group housing with same-sex siblings until PND 48. The study animals were then moved to single housing until after the last behavioral task (see Fig 2).

### Behavioral tasks

In the behavioral tasks, no more than one male and one female rat from each litter were used from each experimental condition. Therefore, no more than eight rats came out of each litter of 12 pups. All behavioral tasks were conducted with white noise playing in the room to inhibit auditory distractions or stressors. Light conditions were different for each task. A light meter was used at the beginning of each daily session to ensure the lighting was consistent across all trials. All rats were placed in a room for 5 minutes prior to initiating the behavioral task to allow habituation to the room change and new conditions. All equipment and transfer cages were wiped down with a 10% vinegar and water solution between rats. Behavioral data was collected using AnyMaze tracking software.

#### Elevated-plus maze

The elevated-plus maze (EPM) task was conducted on PND 50. The arms of the maze were arranged in a plus shape where two opposing arms had high walls, and the other two arms had a slight lip (arms were 11.5 cm wide and 137 cm long). This task was conducted in red-light conditions, measured at an average lux of 19.5 for the open arms, with a range of 16.7–23.6, and an average lux of 0.43 with a range of .37-.50 for the enclosed arms. At the end of the 5-min room habituation, the rat was placed at the center of the elevated-plus-maze facing between two arms. The researcher exited, and the rat was permitted to roam freely for 5 minutes. The distance traveled and the average time spent on each arm were measured. The number of fecal boli deposited on the maze was recorded as well. Time spent in each type of arm was used to indicate anxiety-like behavior [27].

#### Open-field task

The open field (OF) task was conducted on PND 55 or 56. Because many rats were born on the same day, the litters were semi-randomly chosen to run with a four or five-day interval in between the EPM and OF tasks. Of those with four days between EPM and OF, five cohorts were 15-min control animals, and 6 were 180-min experimental rats. There were four 15-min control cohorts in the groups with five days between the two tasks and 2 cohorts in the 180-min experimental groups. The OF apparatus was a white box that measured 103 cm x 103 cm with outer walls that spanned 46 cm high. The rats ran the task in dim white light, with an average lux of 38.5 in the four corners of the apparatus (ranging between 32.1 and 43.2), and an average lux of 50.5 in the center (ranging between 49 and 52.4). After the 5-min room habituation, animals were placed in the center of the OF apparatus to roam freely for 10 minutes. The distance traveled and the amount of time the animal spent in the center of the apparatus versus the perimeter was measured using AnyMaze software. The number of fecal boli left on the apparatus was also recorded. Animals that spent more time in the open center were considered to exhibit less anxiety-like behavior [28].

#### Social interaction

**Fig 3:**
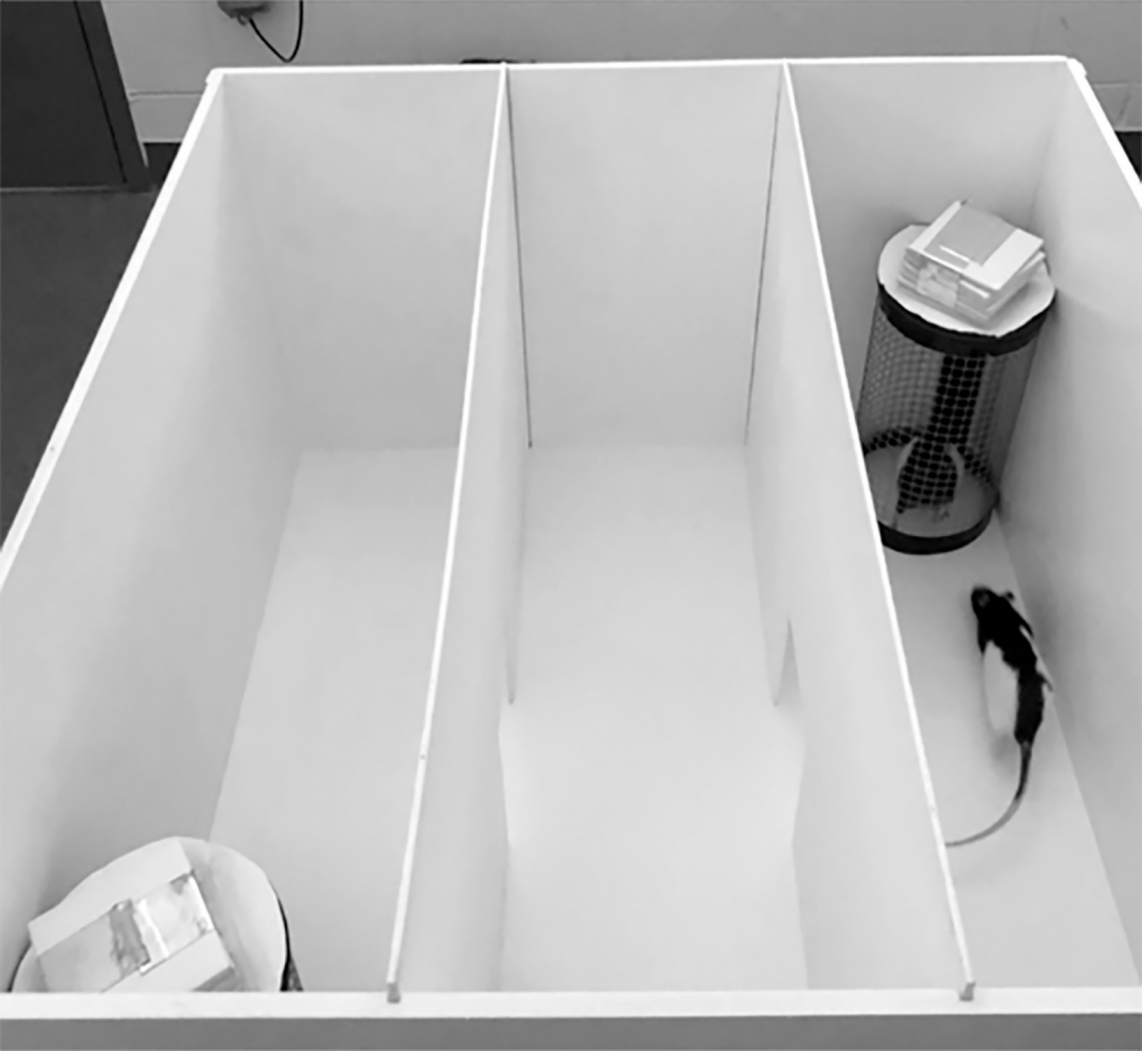
Social interaction and social discrimination apparatus. The apparatus for the Social Interaction (SI) and Social Discrimination (SD) tasks. Image reprinted under a CC BY license with permission from Pamela A. Jackson, 2021.

The Social Interaction (SI) task was conducted on PND 56 or 57 to measure sociability. The apparatus used was the same as the OF task, but the field was divided into three chambers of equal area by adding slide-in walls with small, open doors connecting all three chambers. The task was ran in dim white light (as in OF), with an average lux of 31.8 in the four corners (ranging between 27.2 and 35.5), and an average lux of 40.7 in the center (ranging between 40.4 and 42.4). Two round metal cages were positioned at opposing corners of the apparatus, and the corners alternated within and between rats. One cage held a conspecific rat, while the other cage remained unoccupied (see Fig 3). The conspecific rats used for both the SI and the SD tasks were same-sex and similar in age to the study rats. They were often the extra rats produced by the breeding process but were never from the same litter.

The study rat and the conspecific rat were habituated to the room simultaneously for five minutes before initiating the trial. The conspecific rat was placed in the cage just before the trial began. Then, the study rat was placed at the center of the apparatus to roam freely for 10 minutes. After each study animal completed the task, the apparatus and holding cages were thoroughly wiped down using a 10% vinegar solution. The amount of time the rat spent interacting with either cage was measured by the experimenter in parallel with the AnyMaze software, and the experimenter coding was recorded by the AnyMaze software as well. The experimenter was in a neighboring room viewing the rat via video and pushed computer keys corresponding to interactions with each cage. The more time the study rat interacted with the cage holding the conspecific rat, the higher the rat scored on sociability. The empty cage position and conspecific rat differed between trials and were counterbalanced across conditions within each sex and experimental condition.

#### Social discrimination

The Social Discrimination (SD) task was conducted on PND 57 or 58. The task was utilized as a measure of sociability and memory. The same apparatus and a similar procedure from the SI task were used for SD. The lighting was also the same, with an average lux of 32.2 in each of the four corners (ranging between 27.7 and 35.5), and an average lux of 41.5 in the center (ranging between 39.3 and 43.5). The procedure between the two tasks is dissimilar because both cages in SD housed a conspecific rat. One cage housed the same conspecific rat used during the SI task 24-hours before, making it the familiar conspecific. The other cage held a new, unfamiliar conspecific rat. The placement was counterbalanced across rats and groups. The number of contacts and the amount of time the study rat spent interacting with each conspecific cage within the 10-min period was measured by the human experimenter and the AnyMaze system. Spending significantly more time interacting with the new conspecific rat indicated better recognition of the new versus the familiar rat and a more typical pattern of social behavior.

### Analyses

The data was imported into SPSS from the AnyMaze program. Overall analyses were performed first with all isolation factors included in a 2x2x2x2 ANOVA univariate design: isolation-time (ISO) group (15-min control vs. 180-min experimental), sex (male vs. female rats), presence of dam (dam present vs. dam not present during isolation), and presence of siblings (single-housed vs. group-housed during isolation). If the interpretation of a significant interaction was unclear, the data were analyzed again separately for that factor (e.g., if an interaction with sex was significant, all males and all females were examined separately using a 2x2x2 design). Repeated measures analyses were conducted as well, depending on the task. Only significant findings (*p* < .05) are reported for each analysis. Graphs were produced using Prism software and included the standard error bar.

## Results

### Body weight

All rats of the same sex weighed the same at the beginning of the isolation manipulation. A 2x2x2x2 ANOVA conducted on the body weight data collected on postnatal day (PND) 1 revealed a significant main effect of sex (*F*(1,108) = 6.32, *p =* .013, *η*^2^ = .055), such that male rat pups (*M* = 7.45 g) weighed significantly more than female pups (*M* = 7.05 g) overall. There were no main effects for the ISO group (180 vs. 15 min isolation condition), the presence of the dam, the presence of siblings, or any interactions.

**Fig 4:**
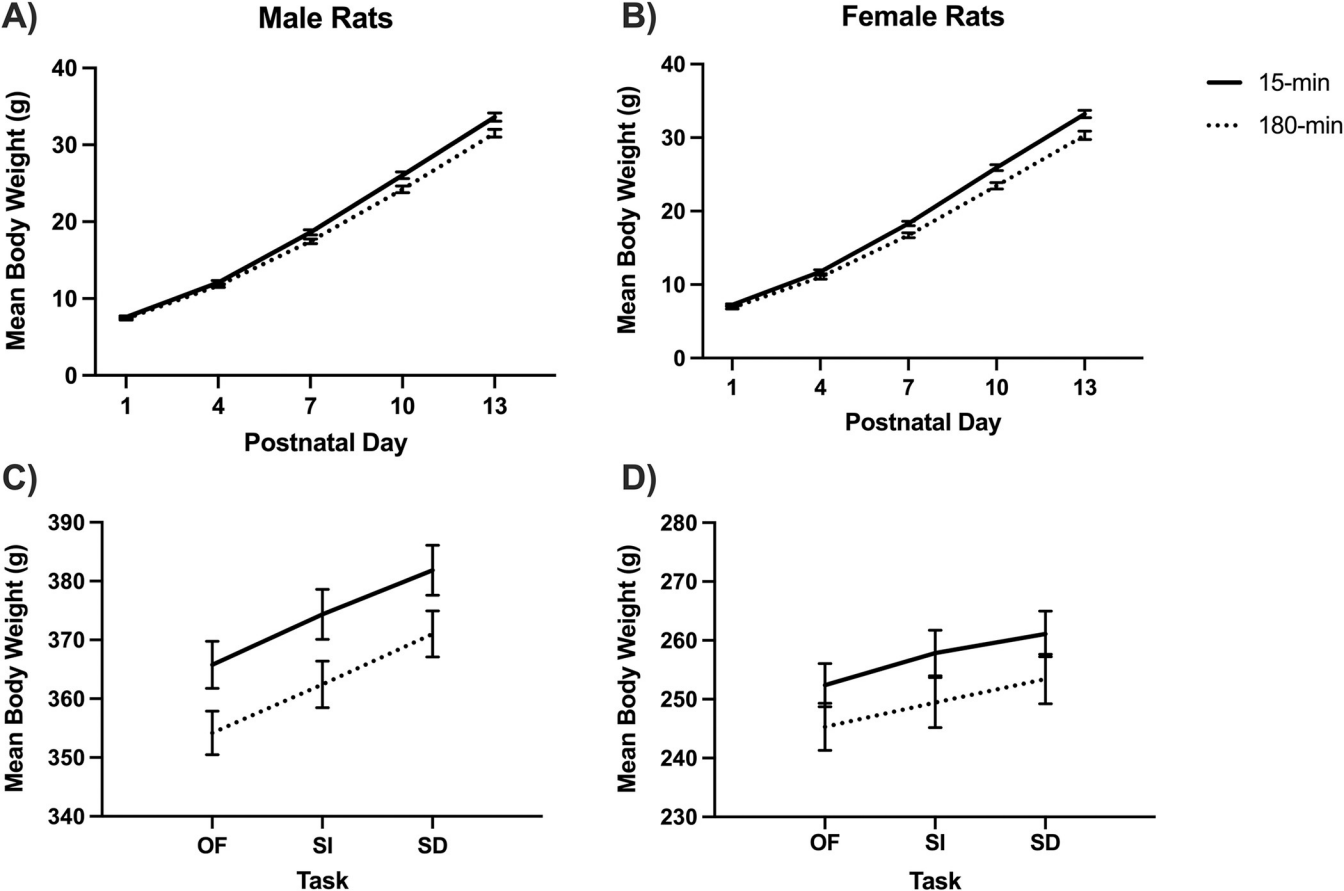
Mean body weight. A & B) The mean body weight in grams on postnatal days (PNDs) 1, 4, 7, 10, and 13 for the control group (15-min isolation) versus the experimental group (180-min isolation) for the male (A) and female (B) rat pups during the isolation manipulation period. Mean body weight across the open-field (OF), social interaction (SI), and social discrimination (SD) behavioral tasks on PND 55–58 for male (C) and female (D) rats. The control and experimental groups differed significantly overall, as well as males versus females, but there were no significant group interactions.

A 2x2x2x2x5 repeated measures ANOVA on body weight data collected on every third day of the isolation period illustrates the effect of social isolation on body weight. All rats gained weight (*F*(4,428) = 9049.19, *p <* .001, *η*^2^ = .988). The main effect of ISO group was significant (*F*(1,107) = 18.85, *p <* .001, *η*^2^ = .150) such that the overall mean weight of the control group was higher (19.44 g) than the experimental group (18.06 g). Additionally, the interaction of PND and ISO group was also significant, indicating that the experimental group (180-min ISO) gained weight slower (see Fig 4A and 4B; *F*(4,428) = 20.88, *p* < .001, *η*^2^ = .163) than the control group (15-min ISO). There were no significant main effects or interactions with sex in this analysis.

On PND 50, rats were placed in the elevated plus-maze (EPM) task for five minutes. A 2x2x2x2 ANOVA of body weight on the day of the trial revealed a significant main effect of sex (*F*(1,105) = 486.63, *p <* .001, *η*^2^ = .823), such that male rats (*M* = 308.44g) weighed more than females (*M* = 224.38 g). The main effect of ISO group was also significant (*F*(1, 105) = 7.33, *p =* .008, *η*^2 =^ .065) where 15-min rats (*M =* 271.57 g) weighed more than 180-min rats (*M =* 261.25 g). There were no other main effects or interactions.

There was a break of 4 or 5 days between the EPM task and the open-field (OF) task. A 2x2x2x2x3 repeated measures ANOVA of body weight on the next 3 days of behavioral data collection for OF, social interaction (SI), and social discrimination (SD) revealed that all rats gained weight across the three days (*F*(2,202) = 418.71, *p <* .001, *η*^2^ = .806), with the means being 304.41 g for OF (PND 55 or 56), 311.02g for SI (PND 56 or 57), and 316.85 g for SD (PND 57 or 58). As expected, male rats (*M* = 368.27 g) weighed significantly more (*F*(1,101) = 835.09, *p <* .001, *η*^2^ = .892) than female rats (*M* = 253.26 g). In addition, the males gained more weight over time (*F*(2,202) = 43.85, *p <* .001, *η*^2^ = .303) than the female rats. Between-subjects effects also showed a significant ISO group difference (*F*(1,101) = 5.80, *p =* .018, *η*^2^ = .054) where the 15-min rats weighed more (*M* = 315.55 g) than the 180-min rats overall (*M* = 305.97 g). See Fig 4C and 4D.

### Isolation manipulation

Pacing (walking in the cell) and grooming (scratching, nibbling, or licking themselves) during the isolation period were chosen as measures of both activity and anxiety. Only the first behavioral measure taken at 10 to 15 minutes into each isolation session was analyzed to avoid bias between the 15-min and 180-min ISO groups. The percentage of sessions on which the behavior occurred was obtained by dividing the number of observations by 14 (total sessions) and multiplying by 100 for each rat.

**Fig 5:**
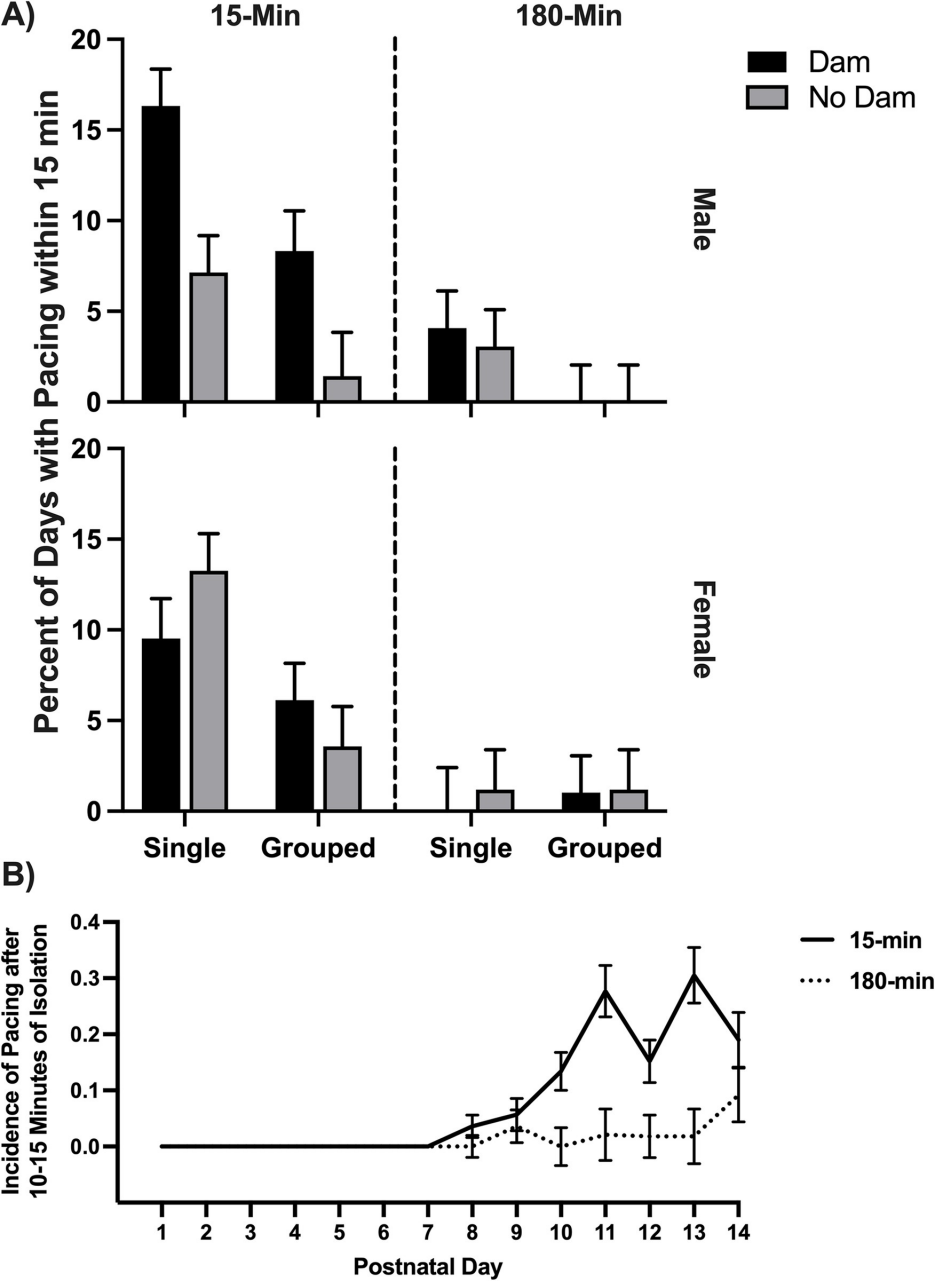
Pacing in isolation. A) Mean percent of sessions on which pacing occurred on the first observation during the isolation period. The interaction of sex by the presence of the dam was significant, as well as the main effect of group. B) The mean incidence of pacing on the first observation across isolation sessions for the control group (15-min) versus the experimental group (180-min). Single = Isolated without siblings; Grouped = Isolated with siblings; Dam = Isolated with the dam; No Dam = Isolated without the dam.

The 2x2x2x2 ANOVA for the percent of days on which pacing occurred revealed several significant findings. The main effect of ISO group was significant (*F*(1,87) = 41.57, *p <* .001, *η*^2^ = .323), such that the control group paced significantly more frequently (8.21%) than the experimental group (1.32%). The main effect of the presence of siblings was also significant (*F*(1,87) = 14.81, *p <* .001, *η*^2^ = .145). The single-housed group had a mean of 6.82% of trials with pacing, whereas the group-housed rats paced during 2.71% of sessions. The interaction between sex and the presence of the dam was significant (*F*(1,87) = 5.28, *p =* .024, *η*^2^ = .057). The data are graphed in Fig 5A. These data show that the male rats housed with the dam on the other side of the barrier in the 15-min groups paced more than the males with no dam present, whereas the female rats paced about the same with or without the dam being present. The effect did not occur in the 180-min groups, probably due to the extremely limited pacing in the experimental groups (attributed to severe arousal). As can be seen in Fig 5A, having the dam present may have reduced anxiety which resulted in increased pacing, but only in the males. The interaction of ISO group with the presence of siblings was also significant (*F*(1,87) = 5.84, *p =* .018, *η*^2^ = .063). The data illustrate that the 180-min rats exhibited very little pacing, but in the 15-min groups, the single-housed rats paced significantly more than those housed with siblings.

As stated above, the 15-min control group paced more than the 180-min experimental group during the isolation sessions. A 2x2x2x2x14 repeated measures ANOVA of the average number of pups pacing each day was conducted as well. It revealed that pacing increased across sessions in general (*F*(13,1131) = 10.17, *p <* .001, *η*^2^ = .105), and this interacted with ISO group (*F*(13,1131) = 6.47, *p <* .001, *η*^2^ = .069). As can be seen in Fig 5B, the rats did not begin pacing until session 8, likely due to the development of locomotor ability [29]. After that point, pacing in the 15-min rats increased, whereas very few of the 180-min rats paced until session 14. The interaction between PND and the presence of siblings was also significant (*F*(13,1131) = 2.06, *p =* .014, *η*^2^ = .023). There was a significant between-subjects effect for ISO group (*F*(1,87) = 41.57, *p <* .001, *η*^2^ = .323), as well as presence of siblings (*F*(1,87) = 14.81, *p <* .001, *η*^2^ = .145). There were interactions between ISO group and presence of siblings (*F*(1,87) = 5.84, *p =* .018, *η*^2^ = .063) as well as sex and presence of the dam (*F*(1,87) = 5.28, *p =* .024, *η*^2^ = .057).

**Fig 6:**
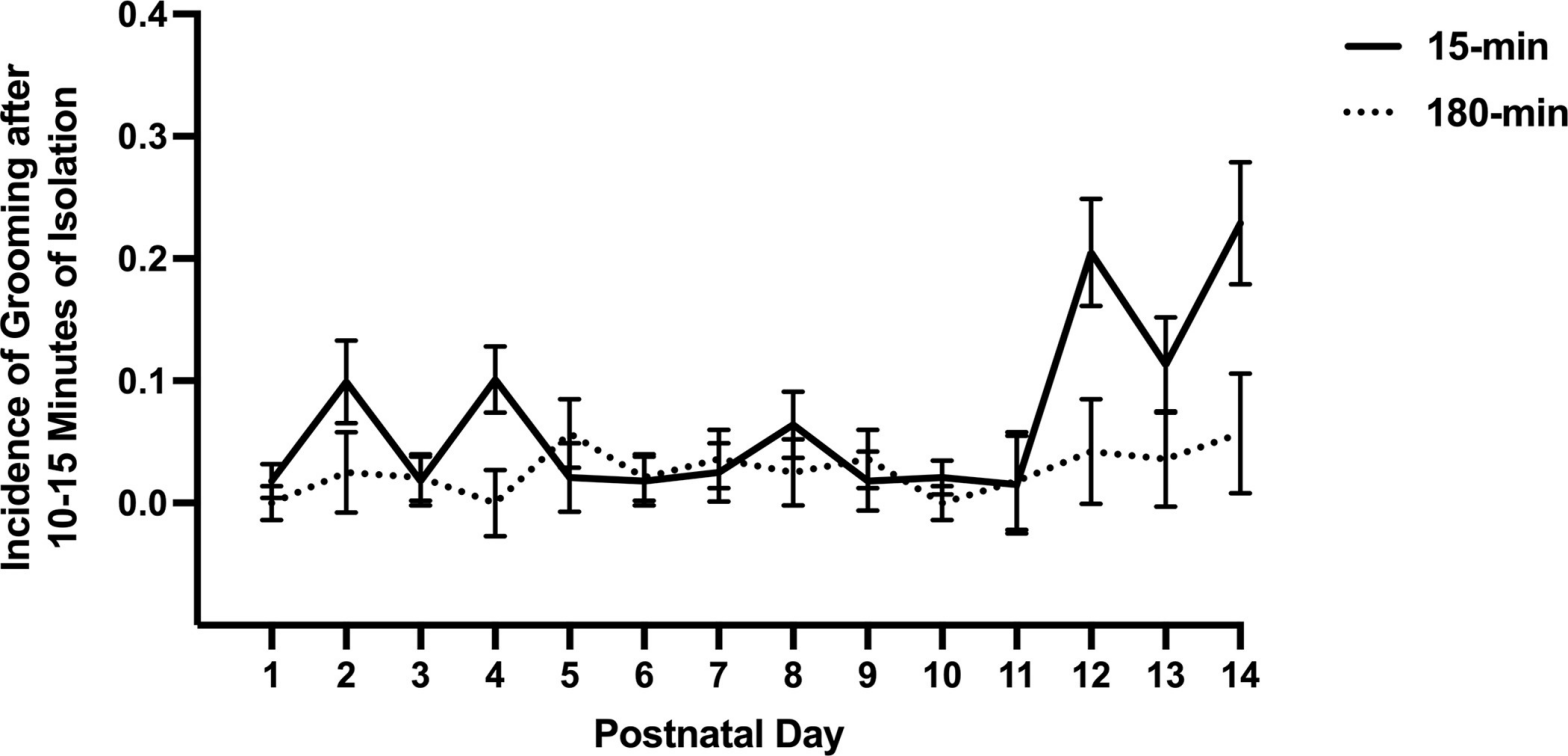
Grooming in isolation. The mean incidence of grooming each day during the first observation of the isolation sessions as a function of the control group (15-min) versus the experimental group (180-min).

As with pacing, grooming was also suppressed in the experimental groups. The repeated measures analysis of percent grooming revealed a significant main effect of ISO group (*F*(1,87) = 18.51, *p <* .001, *η*^2^ = .175). The mean percentage of days on which the rats groomed in the 15-min group was 7.87%, and in the 180-min group, it was 2.65%. The interaction of PND and ISO group was also significant (*F*(13,1131) = 2.56, *p* = .002, *η*^2^ = .029). The data for each session is graphed as the mean percent of pups grooming in the first 10–15 minutes of the isolation. As can be seen in Fig 6, grooming behavior occurred in some rats almost from the first session of isolation. However, at PND 11, the control group began to show an increase in grooming that the experimental group did not.

### Elevated-plus maze (EPM)

**Fig 7:**
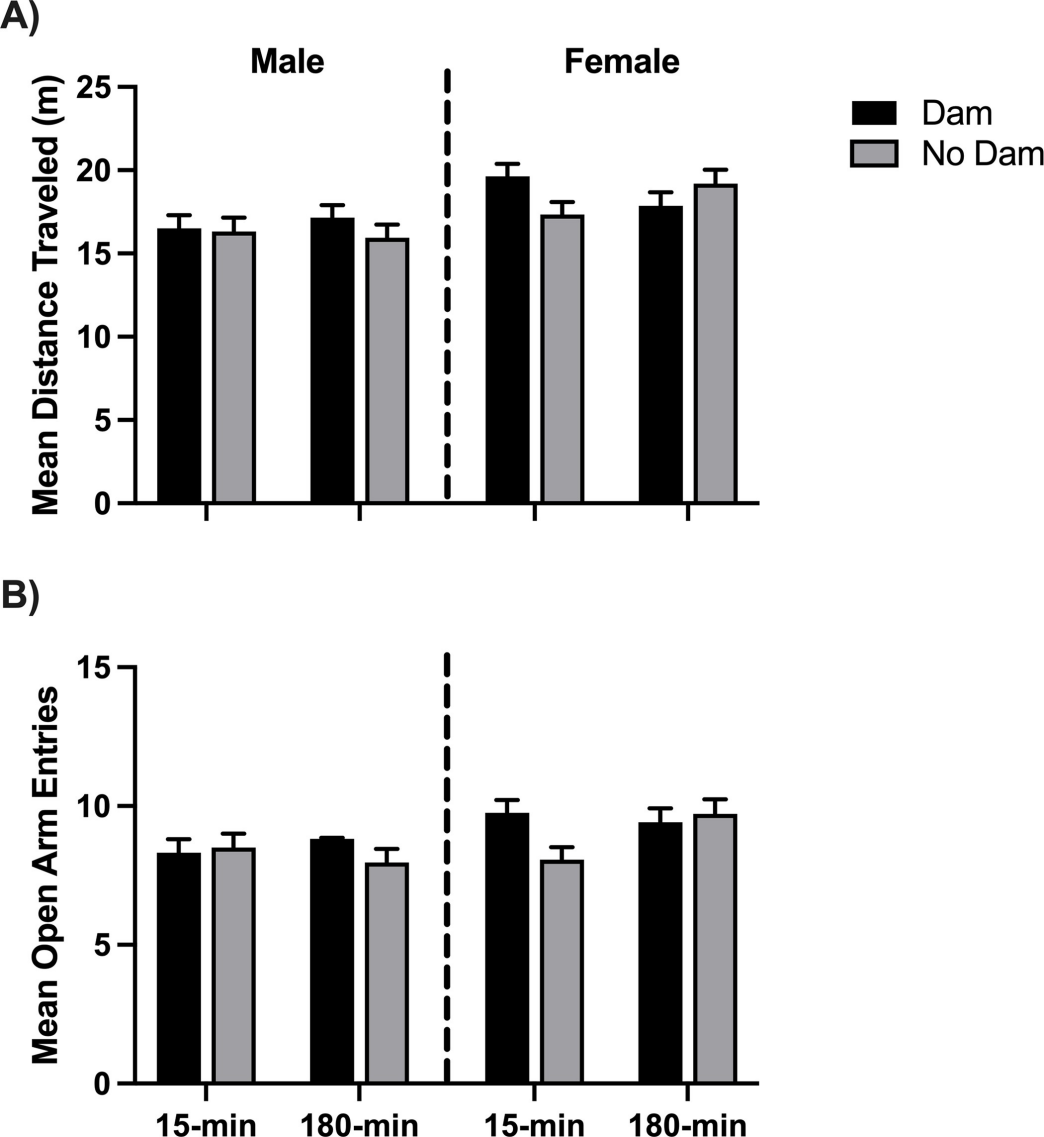
Elevated-plus maze task. A) Mean distance traveled during the elevated-plus maze (EPM) task. The interaction between the presence of the dam and isolation group (ISO) was found to be significant in the female rats. B) Mean entries into open and enclosed arms. The interaction of ISO group and dam present in the female rats was significant. 15-min = Isolated for 15 minutes; 180-min = Isolated for 180 minutes; Dam = Isolated with the dam; No Dam = Isolated without the dam.

The rats’ movements on the elevated-plus-maze (EPM) task were recorded, including time in each zone, zone entries, and distance traveled throughout the 5-minute period. The 2x2x2x2 ANOVA of distance traveled on the EPM revealed a main effect for sex (*F*(1,104) = 13.14, *p <* .001, *η*^2^ = .112). Female rats (*M =* 18.51 m) were significantly more active (traveled further) than male rats (*M =* 16.49 m) on the EPM. There was also a significant interaction between sex, ISO group, and presence of dam (*F*(1,104) = 4.29, *p =* .041, *η*^2^ = .040) shown in Fig 7A. Given that sex was significant, separate 2x2x2 analyses were conducted for the male and female rats. All significant effects disappeared for males. However, a significant interaction of ISO group by the presence of the dam remained for female rats (*F*(1,52) = 5.33, *p* = .025, *η*^2^ = .093). Female rats in the control group (15-min) were significantly more active when the dam was in the adjoining cage during the isolation sessions. The other three groups did not differ from the male rats (see Fig 7A).

A 2x2x2x2x2 repeated measures ANOVA of the open versus enclosed arm entries on the EPM showed that all rats (*F*(1,104) = 131.10, *p <* .001, *η*^2^ = .558) entered the enclosed arms more often (*M* = 10.24) than the open arms (*M* = 7.40). Results also showed a between-subjects effect of sex (*F*(1,104) = 5.93, *p* = .017, *η*^2^ = .054) such that female rats entered all arms more frequently (*M* = 9.24) than male rats (*M* = 8.40). This would be expected, given that the female rats were more active in general. Results also showed a between-subjects interaction of sex, ISO group, and presence of dam (*F*(1,104) = 4.76, *p* = .031, *η*^2^ = .044). Follow-up analyses for each sex showed that both male and female rats entered the enclosed arms more frequently than the open arms. However, the female analysis revealed a significant interaction between ISO group and the presence of the dam (*F*(1,52) = 4.11, *p* = .048, *η*^2^ = .073). When the dam was present during isolation, the female rats in the 15-min group entered arms at about the same rate as the 180-min rats. However, when the dam was not present, the 15-min rats entered the arms less frequently. Overall, the female rats isolated without the dam for 15-min were less active and entered open and enclosed arms less frequently (see Fig 7B). To indicate a change in anxiety the rats should show a significant interaction between entries to the open arms versus the enclosed arms and this did not occur.

Rats typically avoid the open arms on first encounters with the EPM. Rats with higher anxiety-like behavior show increased avoidance of the open arms, which makes the task a widely accepted measure of anxiety [27]. A 2x2x2x2x2 repeated measures ANOVA between time in open arms versus time in enclosed arms showed that the rats overall greatly preferred (F(1, 104) = 52.42, *p <* .001, *η*^2^ = .335) the enclosed arms (*M* = 146.89 s) over the open arms (*M* = 97.25 s). There were no other main effects or interactions.

Fecal boli is another common measure of anxiety [28]. A 2x2x2x2 ANOVA of the number of boli on the maze revealed a significant interaction between sex and the presence of siblings (*F*(1,104) = 4.08, *p* = .046, *η*^2^ = .038). The male rats isolated without siblings (*M* = 2.03) produced more boli than the other groups, suggesting that males isolated alone were more anxious overall on the first day of behavioral testing. The male rats isolated with siblings (*M* = 1.02) and the females with siblings (*M* = 1.12) were very similar, while the female rats isolated alone produced the least amount of boli (*M* = 0.53).

### Open field (OF)

**Fig 8:**
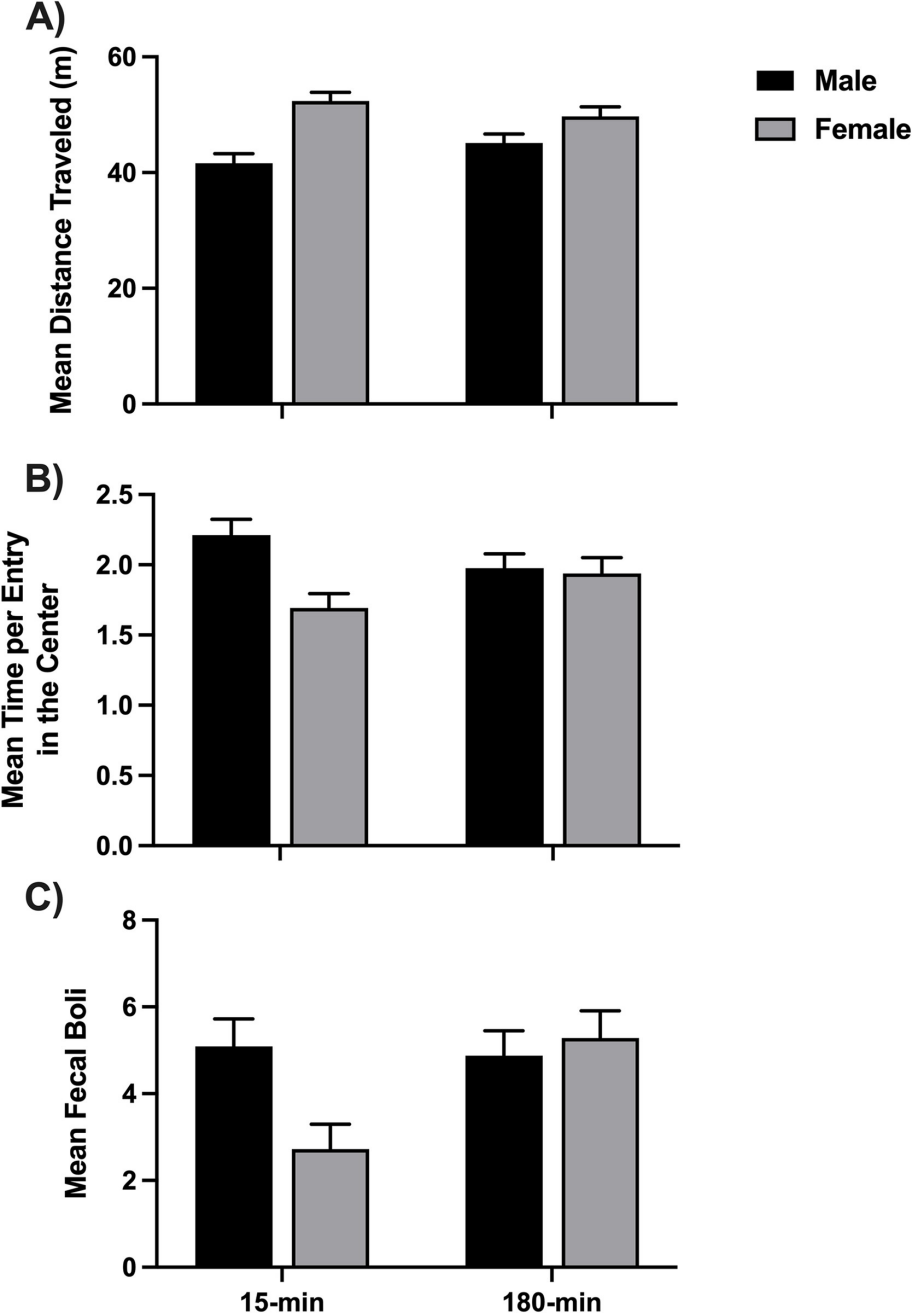
Open field task. A) Mean distance traveled during the open-field (OF) task. The interaction of ISO group and sex was almost significant (*p* = .056). B) Mean time spent per entry to the center during OF. The interaction of ISO group and sex was significant. C) Mean fecal boli deposited on the maze during the open-field task. The interaction of ISO group and sex was again significant. 15-min = Isolated for 15 minutes; 180-min = Isolated for 180 minutes.

On PND 55 or 56, rats were placed in the open field (OF) apparatus for 10 min. A 2x2x2x2 ANOVA of distance traveled revealed one significant effect: a main effect for sex (*F*(1,103) = 22.96, *p <* .001, *η*^2^ = .182). Once again, females (*M* = 51.04 m) were more active than males (*M* = 43.36 m). These data are graphed in Fig 8A.

Given the data showing that female rats were more active than males, we chose not to analyze the more common measure of anxiety, which is the percent time spent in the center of the OF. Instead, we analyzed the mean time per entry to the center of the OF, which would minimize the effect of increased entries by females. All rats avoided the center of the OF consistent with increased anxiety across all groups (overall mean of 5.79% in the center versus the perimeter of the field). The 2x2x2x2 ANOVA of mean time per entry to the center revealed a main effect of sex (*F*(1,103) = 6.60, *p =* .012, *η*^2^ = .060). As might be expected, the less active male rats (*M* = 2.09 s) spent more time per entry than females (*M* = 1.82 s). There was also a significant interaction of sex and ISO group (*F*(1,103) = 4.97, *p =* .028, *η*^2^ = .046). As shown in Fig 8B, there was a sex difference in the 15-min control groups, such that the male 15-min rats were the least anxious and the 15-min female rats avoided the center the most (were more anxious). There was no sex difference in the 180-min groups, and they fell in between the two control groups regarding the amount of time spent in the center per entry.

Finally, a 2x2x2x2 ANOVA of the number of fecal boli in the maze at the end of the trial revealed a significant interaction between sex and ISO group (*F*(1,103) = 5.25, *p =* .024, *η*^2^ = .049). Once again, there was a sex difference in the 15-min groups such that the 15-min females had the least boli and all other groups were equal (see Fig 8C), indicating lower anxiety-like behaviors in the 15-min control females.

### Social interaction (SI)

Social interaction (SI) data was collected on PND 56 or 57 in the OF (after adding walls to create three chambers). Researchers recorded the time the research rat spent interacting with the conspecific cage and the empty cage while simultaneously recording the number of contacts. According to a 2x2x2x2 ANOVA, the study rats and the caged conspecific rats did not differ in terms of body weight.

**Fig 9:**
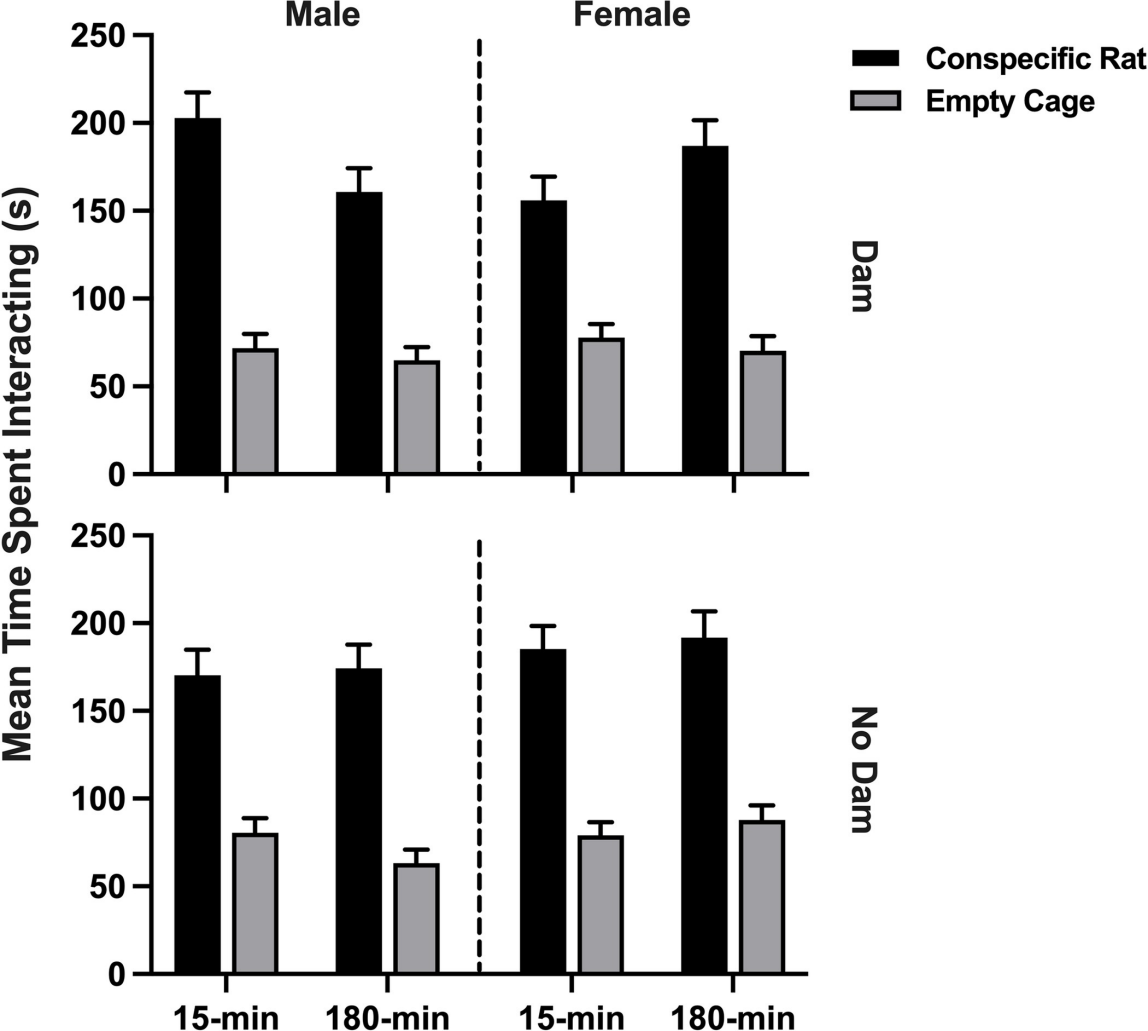
Social interaction task. Average time spent interacting with the conspecific rat versus the empty cage. The interaction of conspecific rat vs. empty cage, isolation group (ISO), sex, and presence of the dam was significant. 15-min = Isolated for 15 minutes; 180-min = Isolated for 180 minutes; Dam = Isolated with the dam; No Dam = Isolated without the dam.

A 2x2x2x2x2 repeated measures ANOVA of the time spent interacting with the conspecific cage versus the empty cage showed that the rats greatly preferred (*F*(1,104) = 313.59, *p <* .001, *η*^2^ = .751) to interact with the conspecific (*M* = 178.52 s) over the empty cage (*M* = 74.46 s). The between-subjects interaction of sex and ISO group was also significant (*F*(1,104) = 5.20, *p* = .025, *η*^2^ = .048). For the male rats, the 15-min control male groups spent more time with both cages than the 15-min experimental rats (*M* = 131.35 vs. 115.82). The female rats, on the other hand, did not show a difference in total time spent with both cages (control *M* = 124.57 vs. experimental *M* = 134.23). There was also a significant four-way interaction (*F*(1,104) = 4.28, *p* = .041, *η*^2^ = .040) between the conspecific versus the empty cage, sex, ISO group, and presence of the dam (see Fig 9). The following two analyses were performed to dissect this interaction.

In order to interpret this four-way interaction, a 2x2x2x2 repeated measures ANOVA was conducted separately for the male and female rats. All rats preferred the conspecific cage over the empty cage in all analyses. There were no other significant effects for the female rats. For males, however, there was a significant ISO group effect (*F*(1,52) = 5.54, *p* = .022, *η*^2^ = .096). The overall conclusion was that the 15-min male rats (*M* = 131.35) spent more time interacting with both the empty and the conspecific cages than the 180-min male rats (*M* = 115.82).

Given that the presence of the dam was a factor in the significant four-way interaction in the overall analysis, separate 2x2x2x2 repeated measures ANOVAs for the dam-present rats and the no-dam-present rats were also conducted. All rats preferred the conspecific cage over the empty cage in all analyses. For the dam present rats, the 3-way interaction of cage, sex, and ISO group was significant (*F*(1,52) = 5.41, *p* = .024, *η*^2^ = .094), as well as the between-subjects interaction of sex and ISO group (*F*(1,52) = 4.56, *p* = .037, *η*^2^ = .081). The 15-min male and 180-min female rats spent more time interacting with the conspecific cage than the other dam-present groups. In the no-dam-present analysis, nothing was significant other than the conspecific vs. empty cage main effect as can be seen in Fig 9. The effects of sex, and ISO group, were absent when the dam was not present during isolation.

The overall conclusion based on all three analyses suggests that all rats exhibited sociability, and all groups interacted with the empty cage for about the same amount of time (*M* = 74.46 s, with a range from 70.4 to 87.7 s). However, male and female rats differed as a function of ISO group if the dam was present during isolation, but they were similar if the dam was not present. The 15-min control males isolated with the dam were significantly more socially oriented than the 180-min experimental male rats. However, the 180-min experimental females were more socially oriented than the 15-min females when the dam had been present. If the dam had not been present during isolation, the male and female 15-min and 180-min groups were about equally social (see Fig 9).

**Fig 10:**
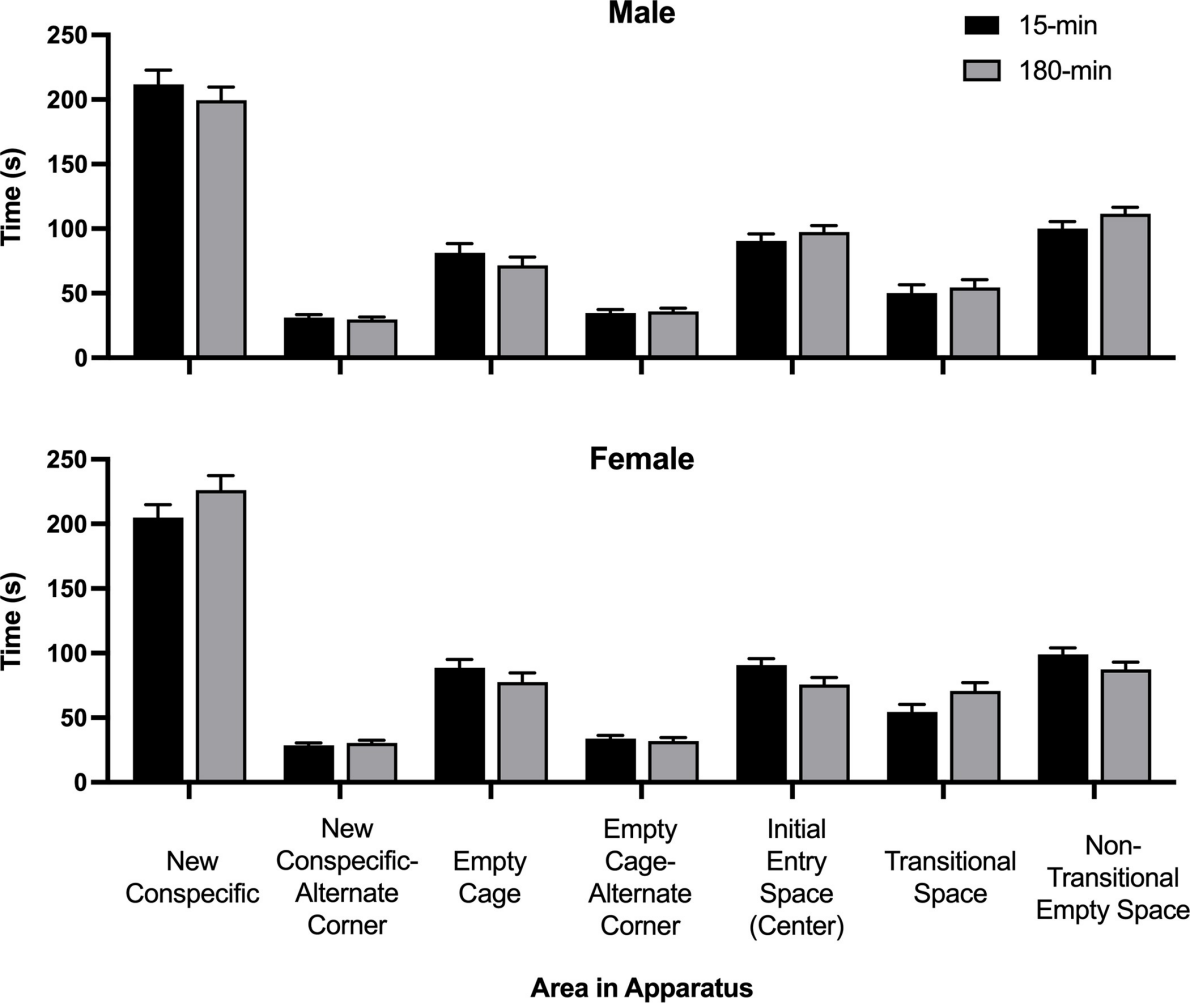
Social interaction location map. Average time spent in each area of the social interaction apparatus as a function of ISO group (15-min = Isolated for 15 minutes; 180-min = Isolated for 180 minutes) and sex (male and female). The field was divided into a 3x3 grid of spaces (see Fig 3). Areas of the grid included the cage with the conspecific (New Conspecific) and the empty corner on the same side but across from the conspecific cage (New Conspecific-Alternate Corner). On the opposite side of the apparatus, the diagonally opposed corner contained the empty cage (Empty Cage), and in the opposite corner, same side as the empty cage, was an empty space (Empty Cage-Alternate Corner). The Initial Entry Space (Center) was the central grid space where the rats were placed in the apparatus at the beginning. The empty spaces between the New Conspecific and the New Conspecific-Alternate corner, and the Empty Cage and Empty Cage-Alternate Corner, were transitional spaces the rats had to travel through to get to the cages (Transitional Space, included 2 spaces). There were also two spaces in the center section, between the corners that held the cages, that were always empty, and the rats did not have to walk through them (Non-Transitional Empty Space).

A 2x2x2x2x2 repeated measures ANOVA of the number of contacts made with the conspecific versus the empty cage was conducted. All rats contacted the cage with the conspecific (*M* = 26.19) significantly more frequently than the empty cage (*M* = 15.71; *F*(1,104) = 160.87, *p <* .001, *η*^2^ = .607). There were no other significant effects. The amount of time spent in each section of the apparatus is depicted in Fig 10.

### Social discrimination (SD)

For social discrimination (SD), rats were placed in the three-chamber sociability apparatus with the same caged conspecific rat from SI in one chamber and an unfamiliar conspecific rat caged in the opposite chamber 24 hours after completing the SI trial.

**Fig 11:**
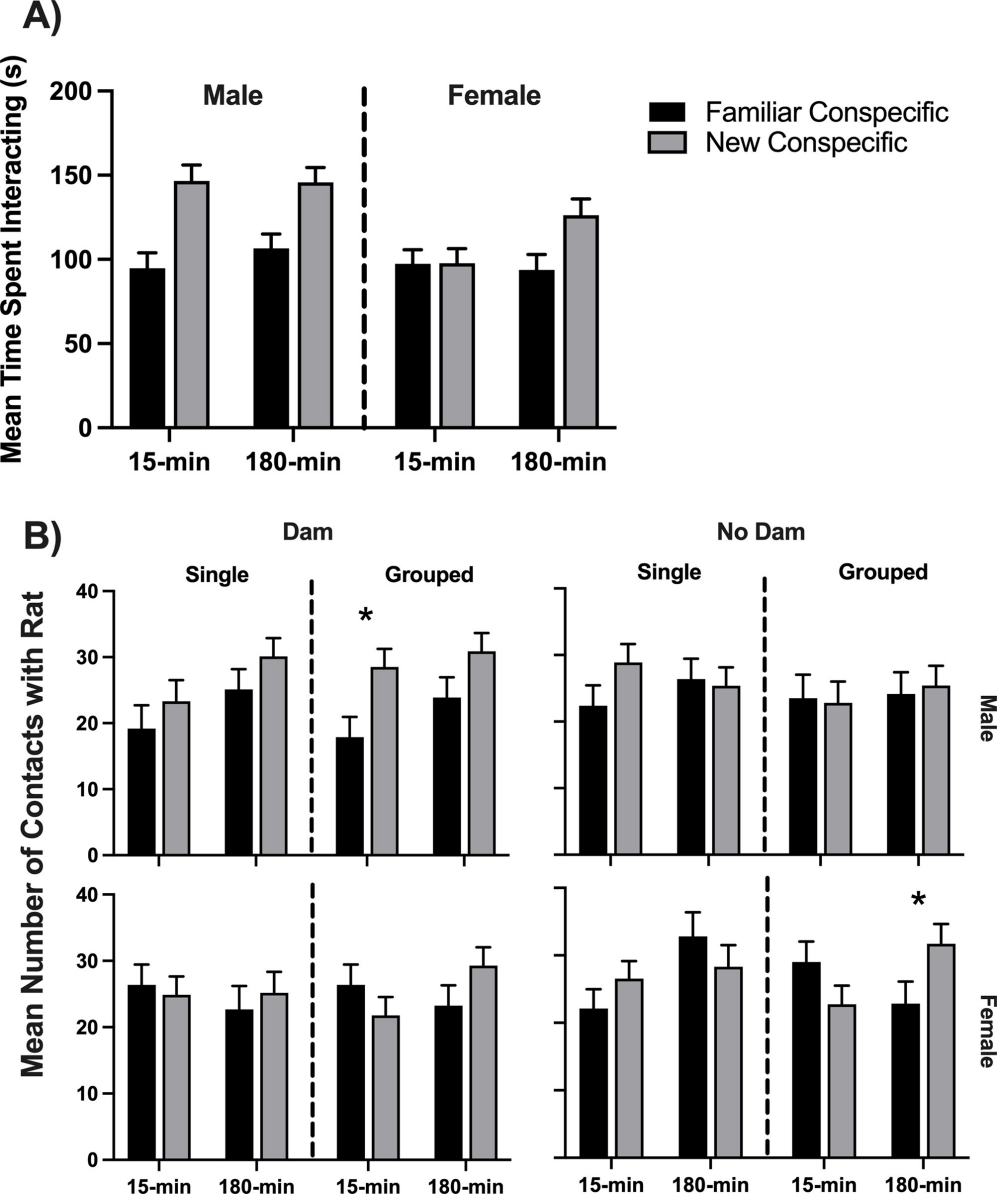
Social discrimination task. A) Average time spent interacting with the new conspecific rat versus the familiar one during social discrimination (SD). The interaction between new versus familiar conspecific and sex was significant. B) The average number of contacts with the familiar rat versus the new conspecific rat in all 16 groups. The males are in the top row, and the bottom row illustrates the females. The asterisk indicates that the pairwise comparison was significant. 15-min = Isolated for 15 minutes; 180-min = Isolated for 180 minutes; Single = Isolated without siblings; Grouped = Isolated with siblings; Dam = Isolated with the dam; No Dam = Isolated without the dam.

A 2x2x2x2x2 repeated measures ANOVA of the time spent interacting with the new conspecific versus the familiar rat revealed that all study rats significantly (*F*(1,103) = 22.19, *p <* .001, *η*^2^ = .177) preferred the new rat (*M* = 129.07s) over the familiar rat (*M* = 98.13s). Furthermore, there was a significant interaction of sex and the new versus familiar conspecific (*F*(1,103) = 4.91, *p* = .029, *η*^2^ = .046). These means are graphed in Fig 11A. In addition, a significant between-subjects effect of sex (*F*(1,103) = 10.19, *p* = .002, *η*^2^ = .090) indicated male rats (*M* = 123.41 s) spent more time with both conspecific rats than females (*M* = 103.78).

Given the significant effects involving sex, a separate 2x2x2x2 repeated measures ANOVA was conducted for the male rats and the female rats. The preference for the new conspecific over the familiar one was the only significant effect in males (*F*(1,51) = 17.11, *p <* .001, *η*^2^ = .251). This effect was also significant for the female rats (*F*(1,52) = 5.10, *p =* .028, *η*^2^ = .089). However, the female rats showed a significant interaction of new vs. familiar conspecific and ISO group (*F*(1,52) = 4.91, *p =* .031, *η*^2^ = .086). As can be seen in Fig 11A, the male rats in both ISO groups spent more time interacting with the new conspecific rat over the familiar one. However, only the 180-min experimental female rats spent more time with the new conspecific, and the 15-min control females did not differentiate between the new and the familiar conspecific rats. The only groups exhibiting a deficit in social discrimination based on time spent with the new conspecific were the 15-min female rats.

The number of contacts to each cage was analyzed with a 2x2x2x2x2 repeated measures ANOVA. Results showed that, as with the amount of time spent interacting, the study rats, on the whole, visited the new conspecific cage (*M* = 26.61) significantly more often (*F*(1,103) = 6.49, *p =* .012, *η*^2^ = .059) than the familiar conspecific cage (*M* = 24.24). The four-way interaction of new/familiar conspecific by ISO group by dam present/not present by single/group-housed was significant (*F*(1,103) = 4.45, *p =* .037, *η*^2^ = .041). In addition, there was a significant three-way interaction (*F*(1,103) = 5.77, *p =* .018, *η*^2^ = .053) of new versus familiar, ISO group, and presence of siblings. The main effect of ISO group was significant (*F*(1,103) = 4.52, *p* = .036, *η*^2^ = .042), with 15-min groups making fewer contacts (*M* = 24.14) than the 180-min groups (*M* = 26.71). The mean data for the number of contacts is graphed in Fig 11B.

Given the significant sex differences in the overall analysis of contacts, a 2x2x2x2 repeated measures ANOVA was conducted separately for the male and female rats. Analysis of the male rats showed that the males visited the new conspecific cage (*M* = 26.92) significantly (*F*(1,51) = 10.03, *p =* .003, *η*^2^ = .164) more often than the familiar conspecific cage (*M* = 22.80). This was the only significant effect in the analysis. Preference for the new conspecific was not true for all groups. The only male group that contacted the new conspecific significantly more frequently than the familiar conspecific was the 15-min control group isolated with siblings and the dam present based on pairwise comparisons (*p* = .004). The 15-min males isolated without siblings but with the dam present appeared to visit the new conspecific more frequently although the pairwise comparison was not quite significant (*p* = .051; see the top row of Fig 11B).

There was no overall difference in the number of contacts to the new conspecific versus the familiar conspecific for the female rats. Analysis of the female rats resulted in a significant interaction of ISO group by the presence of siblings by conspecific (*F*(1,52) = 8.36, *p =* .006, *η*^2^ = .138). In the bottom row of Fig 11B, the standard error bars for female rats overlap for every condition except three. The only female group to show significant recognition that one of the conspecific rats was new by contacting their cage more was the 180-min, dam-not-present, siblings-present group (pairwise comparison *p* = .025; see Fig 11B). All other female groups contacted the new conspecific and the familiar one equally often.

**Fig 12:**
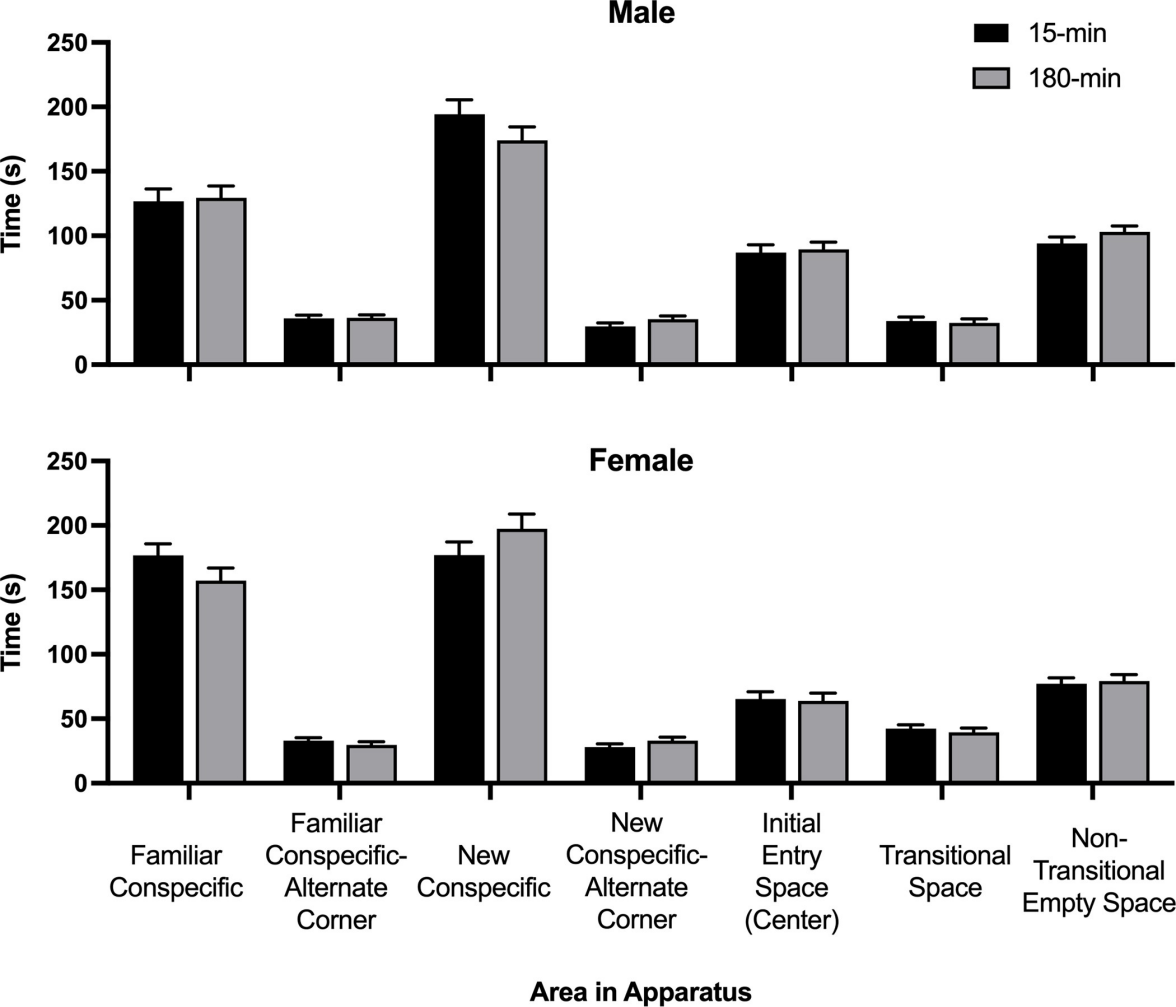
Social discrimination location map. Average time spent in each area of the social discrimination apparatus as a function of ISO group (15-min = Isolated for 15 minutes; 180-min = Isolated for 180 minutes) and sex (Male and Female). The field was divided into a 3x3 grid of spaces (see Fig 3). Areas of the grid included the cage with the familiar conspecific (Familiar Conspecific) and the empty corner on the same side but across from the familiar conspecific cage (Familiar Conspecific-Alternate Corner). On the opposite side of the apparatus, the diagonally opposed corner contained the new conspecific cage (New Conspecific), and in the opposite corner, same side as the New Conspecific cage, was the New Conspecific-Alternate Corner space. The Initial Entry Space (Center) was the central grid space where the rats were placed in the apparatus at the beginning. The empty spaces between the Familiar Conspecific and the Familiar Conspecific-Alternate corner, and the New Conspecific and the New Conspecific-Alternate Corner, were transitional spaces the rats had to travel through to get to the cages (Transitional Space, included 2 spaces). There were also two spaces in the center section, between the corners that held the cages, that were always empty, and the rats did not have to walk through them (Non-Transitional Empty Space).

Overall, most of the rats did not visit the new conspecific rat more frequently, suggesting poor social recognition. The presence of siblings appeared slightly more important for females, whereas the presence of the dam and the siblings may have been somewhat more helpful for males. The amount of time spent in each section of the apparatus is depicted in Fig 12.

## Discussion

The present study sought to compare how the presence of siblings altered the effects of maternal separation on long-term stress. We expected that prolonged isolation, isolation without siblings, and isolation without the dam would lead to adverse development, including reduced body weight, more anxiety-like behaviors, heightened activity, reduced sociability, and a deficit in social memory. However, the results were more complicated due to sex differences and combinations of isolation factors. Two isolation factors that will be considered for the purposes of explanation are stress, which follows the longer isolation time, and comfort, which is higher in rats isolated with the dam or with their siblings.

### Body weight

As expected, body weight for both sexes increased over time, with males typically weighing more than females. Following the hypothesis, rats that were isolated for 180-min weighed less throughout the study compared to rats that were isolated for 15-min. The weight difference was visible from the second day after isolation (Fig 4A and 4B), and it continued for the remainder of the experiment (Fig 4C and 4D). This result is consistent with the findings of Kambali et al. [4], although no significant effects were found for the presence of the dam or the presence of siblings. Given the body weight of the 180-minute rats remained significantly less than the 15-min rats throughout the study, it is likely that the reduced weight is a hindrance in development caused by the stress of isolation rather than an acute physical hindrance to eating. This can be supported by the findings in studies like de Souza et al. [30], which found that maternal separation (MS) can decrease eating behaviors later in life. Another study found that the stress induced by isolation reduced the number of ghrelin receptors, which would reduce the binding of ghrelin, a hormone involved in hunger and anxiety [31]. Although this study did not examine the molecular effects of MS on feeding behaviors, this could be an insightful avenue for future research. In addition, it may help to show the body weight of rats that were never isolated to better compare for the 15-min group, especially considering that a short isolation period followed by high nutritional feeding could present with no weight loss effects [32].

### Isolation manipulation

The combined isolation activity data taken within the first 10–15 minutes of isolation suggests that the longer isolation time was significantly more stressful than the 15-min isolation time in this model. Exposure to novelty typically results in an anxiety-like response that involves increased locomotion, exploratory behavior, and grooming [33]. However, these responses depend on the strength of the arousal elicited, and strong arousal often suppresses all behavior. If pacing during the isolation period is a sign of agitation or mild stress, then the data suggests that the 15-min groups were experiencing mild stress and therefore paced more than the 180-min groups (Fig 5B), who were experiencing severe stress, resulting in very little movement at all. In addition, male rats paced more when isolated with the dam (Fig 5A). However, the 15-min rats paced less when isolated with siblings. These results may fit with the effects of mild stress described in Spruijt et al. [33] and suggest that the dam and siblings influence the comfort level of isolated rat pups. If the dam was nearby but out of reach, mildly stressed male pups increased their pacing as if to try to get to the dam. Females did not present this behavior. Nevertheless, all 15-min rat pups paced more than the 180-min groups, which indicates that the experimental group was strongly affected by the daily 180-min isolation sessions across 14 days.

More grooming observations occurred in the 15-min group compared to the 180-min groups, with grooming rates increasing across isolation days for 15-min groups (Fig 6). Komorowska and Pellis [34] and Spruijt et al. [33] suggested that grooming is a behavior that changes as a function of arousal level in new situations, such that, as habituation occurs, the animal becomes less aroused, and the grooming response lengthens as a function of less stress. This increase in grooming can be seen in both groups but is much more pronounced in the 15-min control groups, suggesting that they began to habituate to the isolation stress, much more so than the 180-min groups. The bottom line is that both measures reported for the isolation period suggest that isolation effectively produced stress to a much higher degree in the 180-min groups, and the pacing data showed evidence from the 15-min group to indicate that the presence of the dam and siblings mitigated that stress or had a comforting effect.

### Behavioral tasks

In the elevated plus-maze (EPM) task, all rats preferred spending time in the enclosed arms, which is typical and commonly attributed to anxiety. No other significant effects were found in arm preference. Female rats, in general, were more active than males, which may suggest mild anxiety, according to Ramos [35]. In addition, the female rats isolated without the dam for 15-min were less active than 15-min females with the dam according to entrances into open and enclosed arms and distance traveled (Fig 7A). This finding suggests that 15-min female rats isolated without the dam had more severe anxiety compared to the 15-min females isolated with the dam. In addition, the male rats isolated without siblings may have also had more mild anxiety according to their number of boli. In general, the males with less comfort, isolated without siblings or without the dam, showed more anxiety, but females with less comfort showed less anxiety in only the shorter isolation time.

Comfort can refer to maternal attention (for the rats near the mother) or being with siblings, and has been shown to increase inoculation [36–38]. According to Qin et al. [37], short periods of maternal separation (1 hour) form a protective factor, called inoculation, against the deleterious effects of stress both behaviorally and physiologically. Maternal licking is a form of maternal attention related to MS [20], but maternal licking has opposing neurological effects on males versus females [14, 22, 23]. These differences are associated with higher anxiety and more sociability in males but lower anxiety and higher aggression in females.

Comfort, and therefore inoculation, would likely be higher in the rats isolated with the dam or with siblings and in shorter isolation times due to lower stress and an earlier reconnection with the dam. Based on these findings, it would be reasonable to expect the presence of siblings and the presence of the dam to increase the inoculation effect through comfort during isolation. The present study supports these assumptions because the 15-min females with less comfort had more anxiety, and males with less comfort also had more anxiety.

For the open field (OF) task, all groups avoided the center of the apparatus, suggesting higher anxiety. In addition, there were sex differences for the 15-minute rats, where the males were less, and the females were more anxious than the other rats, which follows sex differences in inoculation (see Fig 8B). These findings support the idea that the 15-min rats were more inoculated since inoculation would produce more anxiety in females but less anxiety in males. The 15-min female rats also had the least boli despite presenting the most anxiety-like behavior (Fig 8C). Since increased boli generally presents with mild as opposed to severe anxiety [28], these findings may suggest that the female 15-min rats were severely rather than mildly anxious. These results also support the findings of Kambali et al. [4], who only tested males and found increased anxiety in the longer isolation group. Again, female rats were more active than males (Fig 8A), but no differences were found between other conditions.

In the social interaction (SI) task, all rats exhibited sociability and preferred the caged rat over the empty cage (Fig 9). For the rats isolated with the dam, males had more sociability in the control group than the experimental group, but females had more sociability in the experimental group than the control group. In the social discrimination task (SD), males were more sociable than females overall, but this appears to have been because the female 15-min rats spent less time interacting with the new conspecific. Sex differences in inoculation support our findings where the more inoculated rats, isolated for 15-min with the dam, had opposite sociability. Males who were more inoculated spent more time interacting with the conspecific rats, and more inoculated females spent less time interacting with the conspecific rats.

The remaining SD results were more complicated than expected. The female rats in the 15-min group failed to show a preference in terms of time spent with the new conspecific rat versus the familiar rat (Fig 11A). This group also showed decreased sociability, possibly due to high anxiety-like behaviors and increased aggression toward both rats. The shorter isolation group was likely more inoculated, implying more aggressive behavior in females. Increased aggression would likely mean less time spent with either conspecific rat producing floor effects within the 15-min females, both of which were found in the SD data. However, overall, the rats did successfully prefer the unfamiliar rat, which did not fit with previous studies. One reason may be that the present study had a 24-hour delay between initial exposure to the familiar conspecific in the SI task and the recognition test in the SD task. Lukas et al. [14] found decreased memory after 60 minutes and no evidence of memory after 120 minutes. There could be many reasons behind why the current rats still managed to remember the rat despite previous studies, but it could be that sleeping overnight increased their memory [39].

The number of contacts the rat had with the familiar versus new rat found more group differences (Fig 11B). For the male rats, only the 15-min, dam-present, siblings-present group showed good recognition, the group with the highest comfort and mildest stress, as follows the inoculation hypothesis (see Fig 11B). Since inoculation has been shown to increase myelination and cortical volumes [37], shorter isolation time, isolation with the dam, and isolation with siblings should increase memory. These results matched previous studies where isolation with the dam increased social discrimination.

The females had more conflicting results. The number of contacts data for the females showed that only the 180-min, dam-not-present, siblings-present group visited the new conspecific cage more than the familiar one (exhibited good memory; Fig 11B). While improved memory was expected for the sibling-present group, it was not expected for the longer isolation or the dam-not-present group. This may have been because the 15-min, dam-not-present group had decreased sociability possibly due to inoculation, meaning less time spent to memorize the familiar rat. These findings did not necessarily match previous studies where the rats under the longer isolation time failed to discriminate between the new and familiar rats [14].

### Limitations

A notable limitation of the present study is that all rats received at least a partial isolation period as infants. The data may have been easier to interpret had we included a control group with no maternal separation at all (a group that only experienced separation for standard animal care). A no-isolation control group may have provided an additional no early handling (EH) comparison, especially for EPM and OF, to determine if all rats in the maternal separation groups were highly anxious, not just those with a daily isolation period, even if only 15-min, because in the Jin et al. [3] study, anxiety was much lower in the unhandled rather than the EH control. Other studies with unhandled control groups found clearer results in anxiety, memory, and activity tasks [2, 4, 10, 14, 17]. However, without controlling for human touch, they could not dissociate the impact of human touch from MS.

The study would also benefit from a larger sample size, allowing for more accurate results, especially for comparing all four conditions simultaneously. Another limitation is that the 180-min rats had decreased body weight caused by the longer isolation (either due to stress or a lack of access to milk), but their decreased body weight continued into the behavioral tasks. The decreased body weight may have caused neurological or psychological changes separate from, or in addition to, isolation stress, resulting in these behavioral differences [31, 32]. Future studies on the impact of MS and SS mechanisms that reduce weight loss may shed more light on our results.

### Future directions

Because the maternal response may alter the effects of early stress, it would help to record the ultrasonic vocalizations during isolation and view the amount of licking within thirty minutes to an hour after the dam is reunited with the pups. The behavioral measurement for that study would help assess which factors contribute to the differences in adolescent behavior, especially if the dam’s preference for pups of each condition could be measured. In addition, licking and vocalizations would allow for comparing the amount of stress and care. Higher maternal care may result in more resilient rats despite higher stress levels.

Neurobiologically, both stress and maternal care are under the control of hypothalamic signaling to the pituitary gland. Specifically, the Hypothalamic-Pituitary-Adrenal (HPA) axis is the primary stress response system in animals. This multi-tiered feedback system involves corticotropin-releasing factor (CRF) that is released from the hypothalamus signaling adrenocorticotropic hormone (ACTH) release from the anterior pituitary, ultimately culminating in the release of corticosterone (CORT) from the adrenal glands [40]. This system works via negative feedback loops and can become dysregulated during chronic stress conditions, especially early life stress [41]. Thus, future studies could benefit from a time course analysis of CRF, ACTH, and CORT in these animals to begin to understand the mechanisms of behavior change observed in this study.

Additionally, oxytocin, a hormone known to be involved in social attachment, is produced in multiple regions of the hypothalamus and released from the posterior pituitary gland directly into the bloodstream [42, 43]. In order to specifically probe some of the sex effects found in the present study, the concentration of oxytocin receptors throughout the brains of both the male and female subjects should be quantified to determine if these levels align with the degree of expected inoculation, the observed maternal licking, or maternal and sibling comfort during isolation [14, 22, 23]. By doing so, insight may be gained into how these neurobiological mechanisms produce inoculation and how comfort could be an important factor of stress inoculation.

## Conclusion

Maternal separation and sibling separation can have developmental impacts in rats. The results of the present study imply that isolation stress in infancy depends on the degree of inoculation. Inoculation describes how mild stress, especially if combined with comfort, can increase resilience to future stress [38]. Inoculation is more likely in rats with a shorter isolation time and more comfort [20, 36–38]. The effects of inoculation and stress are different depending on sex [14, 23, 44]. For example, female rats who were likely more inoculated had decreased sociability compared to male rats with increased sociability. Female rats also had increased anxiety-like behavior in groups likely more inoculated, whereas males had a decrease in groups likely more inoculated. These results show that the shorter isolation group differed in males and females based on comfort conditions, including the presence of the dam and siblings. However, the longer isolation yielded more indicators of stress in the rats but did not vary based on comfort conditions. These results indicate that comfort helps to mitigate the long-term effects of stress during infancy, but it is limited by the amount of stress. This fills a gap in the research from studies that did not directly compare MS, SS, and isolation time. It also more thoroughly expands upon important sex differences in early isolation. In addition, the present study found an overall reduction in body weight in the longer isolation condition that remained throughout the rats’ lifetime. Lower body weight, especially prolonged, seriously impacts human cognitive development [13]. These results have particularly important implications in the care of infants in foster care [5, 6] and premature infants in neonatal intensive care units [24], where similar forms of isolation occur. The results may also apply to other forms of stress during early development [13, 16]. These results indicate that the potentially detrimental effects can be prevented with forms of comfort, particularly a caregiver or siblings.

## Supporting information

S1 Data (XLSX)
